# Extra-Corporeal Membrane Oxygenation Cadaver Donors: What about Tissues Used as Allografts?

**DOI:** 10.3390/membranes11070545

**Published:** 2021-07-19

**Authors:** Gregorio Marchiori, Matteo Berni, Giorgio Cassiolas, Leonardo Vivarelli, Nicola Francesco Lopomo, Milena Fini, Dante Dallari, Marco Govoni

**Affiliations:** 1 Surgical Sciences and Technologies Complex Structure, IRCCS Istituto Ortopedico Rizzoli, Via di Barbiano 1/10, 40136 Bologna, Italy; gregorio.marchiori@ior.it (G.M.); milena.fini@ior.it (M.F.); 2 Medical Technology Laboratory Complex Structure, IRCCS Istituto Ortopedico Rizzoli, Via di Barbiano 1/10, 40136 Bologna, Italy; matteo.berni@ior.it; 3 Department of Information Engineering, University of Brescia, Via Branze 38, 25123 Brescia, Italy; g.cassiolas@unibs.it (G.C.); nicola.lopomo@unibs.it (N.F.L.); 4 Reconstructive Orthopaedic Surgery and Innovative Techniques—Musculoskeletal Tissue Bank, IRCCS Istituto Ortopedico Rizzoli, Via G.C. Pupilli 1, 40136 Bologna, Italy; dante.dallari@ior.it (D.D.); marco.govoni@ior.it (M.G.)

**Keywords:** extracorporeal membrane oxygenation, allografts, transplantation, cadaver donors, preclinical studies

## Abstract

Several studies demonstrated the efficacy of *post-mortem* extracorporeal membrane oxygenation (ECMO) on donors in preserving organ function addressing organ transplantation. Nevertheless, no common and shared evidence was reached about the possibility of using ECMO donors in tissue harvesting. Therefore, this work aimed first to review the current scientific literature about ECMO donors, and then to focus on the use of ECMO tissues as allografts, mainly addressing musculoskeletal tissues, which are of the most interest for reconstruction. A search was conducted on the current scientific literature, focusing on the keywords “ECMO” and “Donor”. Several online databases were used, including PubMed, Scopus, and Web of Science. From the preliminary search, 478 articles were obtained, out of which 173 specifically reported the use of ECMO for donation and transplantation purposes. Literature reported extensive analyses of ECMO organs—overall from the abdomen—both in pre- and post-transplantation studies. On the other hand, ECMO tissues were explanted only in a very limited number of cases; moreover, no information was referred about their status and use. A revision of the current scientific literature highlighted the lack of information concerning ECMO tissues and the necessity to perform preclinical, ex vivo studies to compare allografts from ECMO donors, with respect to standard donors, and, thus, to verify whether they can be harvested and implanted safely and with efficacy.

## 1. Introduction

In the development and validation processes related to novel drugs and medical devices, it is standard practice to pass through in vitro/ex vivo assessments and in vivo animal models before moving on to clinical trials, to demonstrate the overall safety and expected benefits of the products. Concerning organ transplantation, the process may be different due to the urgency of the life-saving treatment and the persistent mismatch between supply and organ demand [1]. Indeed, these needs have been pushing to obtain new kinds of donors directly in clinics. Although clinical reports showed promising results, preclinical models remain important to optimize the procedure [2]. Further, pretransplant biopsies and analyses are fundamental to support clinicians in assessing the quality of the tissues [3].

**Figure 1:**
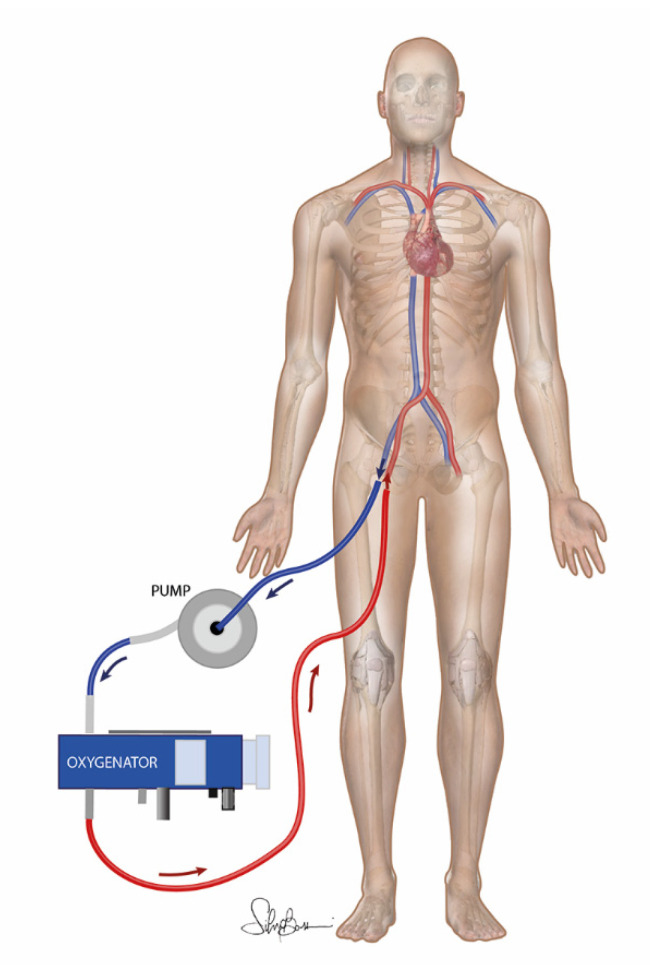
Sketch of the ECMO abdominal recirculation circuit on cadaver donor.

In this perspective, although extracorporeal membrane oxygenation (ECMO, Figure 1), also known as regional perfusion, recirculation, or abdominal oxygenated recirculation [4], is a pivotal tool to support critically ill patients worldwide, *post-mortem* ECMO represents a promising solution and a viable option to expand the organ/tissue donation pool [5,6]. Therefore, as reported by Ortega-Deballon et al. (2015), many countries worldwide have explored the option of donation after circulatory death (i.e., donors after circulatory death—DCD) with the use of extracorporeal perfusion or in situ cooling protocols [7]. When cardiac death occurs in the hospital, DCD is defined as controlled (cDCD), since a decision has been made to withdraw life-sustaining treatments. In this scenario, consent for organ/tissue donation is always obtained by relatives before ECMO procedures begin. Otherwise, in the uncontrolled DCD (uDCD), death is unexpected and generally occurs out of the hospital. Therefore, cannulation and organ preservation with ECMO protocols or in situ cooling can generally begin after hospital arrival, where life-supporting devices are available. For uDCD, consent requirements for donation and organ preservation vary by region or country and may occur before or after cannulation [7]. However, considering that ECMO procedure is an advanced and expensive technology [8], and the allocation of the organ(s) is urged by starting extracorporeal oxygenation strategies [9], it would be more appropriate to acquire the informed consent before cadaver donor cannulation. Further worldwide standardisation of uDCD guidelines is needed to achieve long-term success and widespread implementation of strategies aimed at expanding the number of eligible organ/tissue donors. Indeed, thanks to the ability of providing normal perfusion and oxygenation, ECMO procedure may regenerate cellular energy substrates and, therefore, it has the potential to improve viability of subsequently transplanted grafts [1,10,11]. Moreover, besides its reconditioning effect, ECMO allows organ function to be assessed prior to transplantation. Concerning tissue assessment, ECMO also confers the possibility of conducing intraoperative biopsies, which provide the chance to use reliable grafts [12,13]. However, treatment with ECMO also reported several side effects, such as blood cell consumption and activation [14], and risk of injury inherently related to the reperfusion process [15].

Although a lot of information is available about organs in their entirety [16], tissues are worth investigating as well for two fundamental reasons: (1) to increase and improve the actual knowledge on ECMO organs, since an organ is a complex system consisting of various tissues and the tissue functional state should allow the organ to operate high-level tasks at the best of its potentiality; (2) to reveal ECMO donors as potential new sources also for the banks of tissues, because single tissues can be harvested and used as allografts in reconstructive surgeries, e.g., valves in the heart [17], cornea in the eye [17], and ligaments/menisci in the knee [18,19].

Regarding musculoskeletal allografts, as reported by several authors [20,21,22], bones and their soft tissue components, such as tendons and ligaments, are among the most frequent tissues transplanted worldwide (over two million grafting procedures per year), coming right after blood transfusion. The increasing number of grafting procedures performed every year is related to the ageing population, especially to the fastest-growing segment of individuals over the age of 65. Compared to the USA, where each year more than 900,000 allografts are used [23], in Europe, the trend of the allogeneic tissue transplant (normalised for the total population by country) is slightly lower, although musculoskeletal tissue demand is continuously growing. Some examples are listed, as follows: in France, from 2012 to 2016, the use of allogeneic long bones, femoral heads, and ligaments/tendons increased by 8.3%, 50.8%, and 316.2%, respectively [24]; in Germany, more than 30,000 allogeneic bone transplantations are performed every year [25]; in Italy, more than 10,000 allogeneic locomotor tissues are distributed per year, about 6000 of them by the Musculoskeletal Tissue Bank of the IRCCS Istituto Ortopedico Rizzoli (Musculoskeletal Tissue Bank internal data sources).

Accordingly, the objective of this paper is twofold: the first aim was to conduct a review of the current scientific literature to shed light on ECMO donors, and on the possibility of harvesting and testing ECMO tissues, whereas the second goal was to suggest indications to support the development of novel preclinical/preimplant studies, focusing in particular on musculoskeletal tissues, because these are the most diffused as allografts [26,27].

## 2. Materials and Methods

An in-depth search was conducted on several online databases including PubMed, Scopus, and Web of Science, with no restriction on date of publication up to the end of 2020.

During the first search, the keywords “ECMO” and “donor” were used. In the following screening phase, papers where perfusion was (i) directed to the recipient and not to the donor, (ii) conducted ex vivo (i.e., on the organ isolated from the donor before transplantation, “topical ECMO”), (iii) directed on a specific organ, or (iv) discussed only relatively to the determination of death for donation purposes, were excluded.

**Figure 2:**
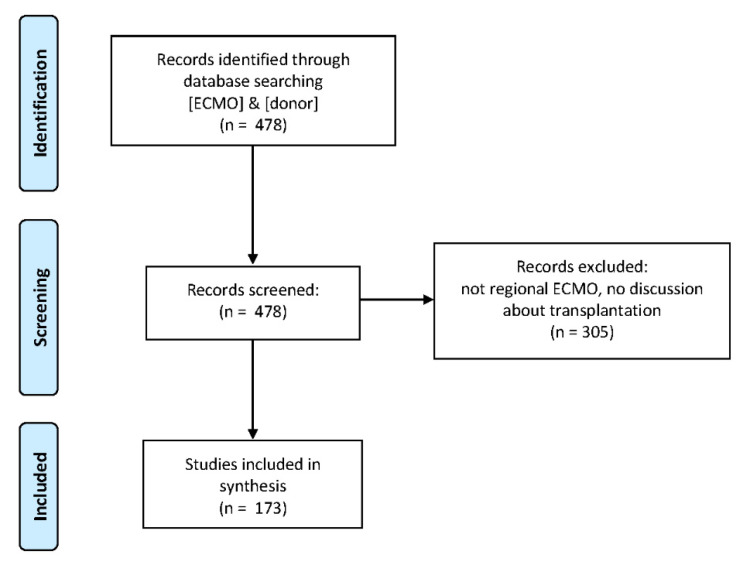
Flowchart showing study identification, screening, and inclusion.

A second screening with the same requirements was finally performed on the referenced articles to complete the list. A flowchart is shown in Figure 2.

Analysis was performed by two separate reviewers, who checked the inclusion/exclusion criteria on each paper, first screening the abstract, and then the main text. The review focused on the typology of donated organs/tissues and the relative performed assessments.

The complete list of included papers in the synthesis is provided in the Supplementary Material, such as Table S1.

## 3. Results

**Figure 3:**
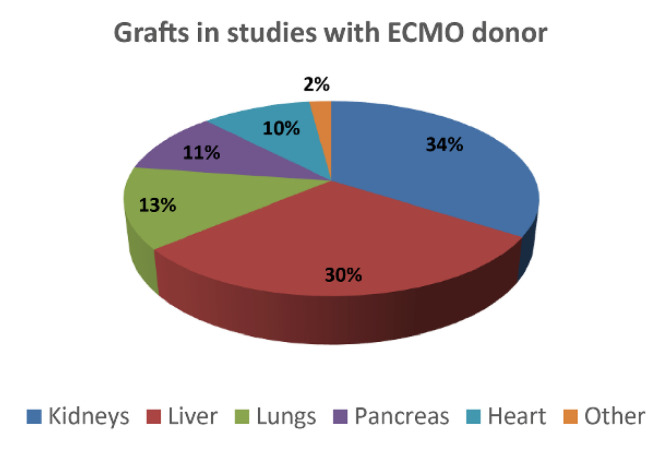
Classification of the identified studies by considering the organ.

In the first phase, 478 studies were identified as possible candidates in this review. After the screening, 173 studies were selected for the synthesis (Figure 2), from 1963 to the end of 2020. First, they have been classified by considering the typology of the investigated tissues and organs (Figure 3).

The majority of the studies pertained to the abdominal organs—in particular, kidneys (34%) [28], liver (30%) [29], and pancreas (11%) [30]—followed by the thoracic organs—lungs (13%) and heart (10%) [12]. Only 2% of the studies involved the harvesting of grafts pertaining different anatomical regions, including cornea [13,17,31], gall bladder and choledochus [32], osteotendinous tissue [13,31], bowel [33], and vascular tissue [13]. A study reported the harvesting of even an entire limb (actually a “topical” ECMO) [34].

**Figure 4:**
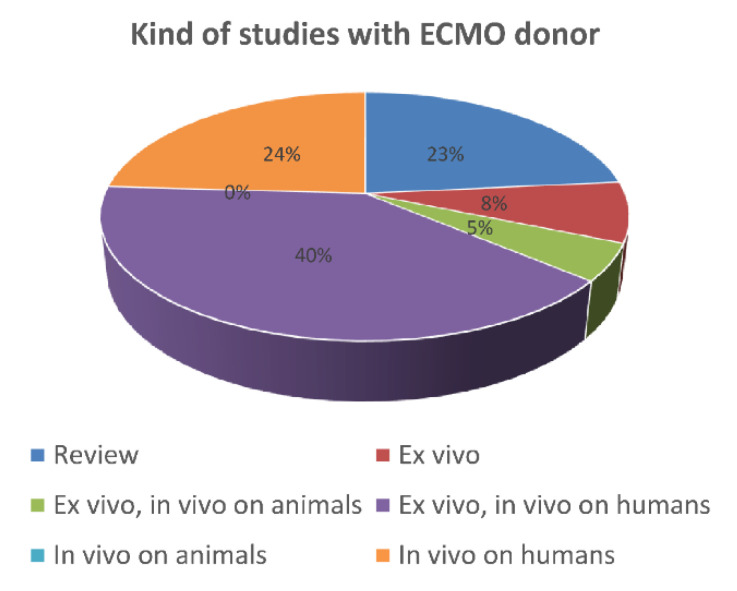
Type of study including ECMO donors.

As shown in Figure 4, the studies were then classified as “review”, “ex vivo”, “in vivo on animals” (i.e., preclinical studies), and “in vivo on humans” (i.e., clinical studies). In particular, ex vivo refers to assessments performed on tissues in experimental conditions outside the organism, with the minimum alteration of natural conditions. This approach implies that ECMO was performed on the donor, but all the analyses were realized outside the donor and the recipient.

Reviews represented 23% of the studies (e.g., in Christopher et al. [16]).

Ex vivo experiments without a transplantation phase—in which the graft was harvested from an ECMO donor and assessed, but not implanted in a recipient—pertained to 8% of the studies. Only one of these works treated human donors [1], while the remaining regarded animal donors [35]. The reported approaches highlighted the importance of preserving human grafts for transplant at the most.

On the contrary, ex vivo evaluations combined with analyses on transplanted animal recipients pertained to 5% of the studies (e.g., in Kerforne et al. 2019 [2]). Those studies performed on transplanted human recipients, relating graft and patient status, were the 40% (e.g., in Farney et al. [36]) and, therefore, the most important group.

Studies were labelled as “pure” in vivo (24%) when focused on the graft functioning inside the recipient and/or on recipient survival. These studies were carried out directly on humans in the form of retrospective studies [37], case reports [38], or clinical trials [39].

Regarding the animal studies, the 87% treated porcine, two bovine [40,41], one canine [41], and one rodent species [42]. The porcine preclinical model is the most diffused one due to size, anatomy, and cardiovascular physiology of the animal, very similar to those of human beings [43].

Most ex vivo investigations pertained to gross evaluation, gas/blood, and tissue biopsy analyses. These studies were performed in preimplant conditions to assess graft acceptability [3,44], or in a postimplant phase to understand malfunctions and/or complications [45,46], or in both [36].

**Figure 5:**
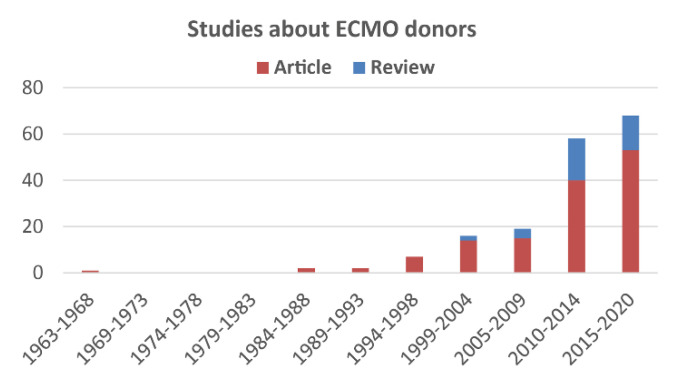
Studies about ECMO donors published from 1963 to 2020.

Despite the fact that experiences on ECMO donors are growing, to such an extent that various reviews on the theme have been recently published (Figure 5), only results about a reduced number of organs have been presented.

## 4. Discussion

Although literature agrees in giving ECMO the potentiality of expanding the overall number of available donors—among the found studies, only one, on rodents, did not find a positive effect of perfusion [42]—ECMO procedure is not without its own drawbacks. Nosocomial infections occurring during ECMO support or related to ECMO devices have already been reported [47]. Thus, to avoid possible contamination of donors’ tissues and organs, a systematic blood culture and intravascular extremity cannula culture are needed and may help to verify device-related ECMO infection. Another potential issue to address is the risk of blood coagulation during the treatment. Indeed, blood proteins accumulate on the circuit membranes, causing membrane fouling, promoting the platelet and leukocyte aggregation to the membrane surfaces. Although heparin-coated membranes are used in many ECMO oxygenators, this solution does not seem to eliminate the risk of coagulation [48]. Thus, the potential formation of blood clots could reduce the oxygenation efficiency during the perfusion procedure and initiate the ischemic cascade in tissues and organs. Hence, an accurate analysis of all the monitored parameters during ECMO support, including also (i) mean perfusion time, (ii) time between donor death and beginning of the ECMO procedure, (iii) arterial and/or venous oxygen saturation, (iv) types of ECMO used (veno-venous or veno-arterial), and (v) type of membrane in the ECMO device (e.g., coating and/or material), is needed to properly evaluate and further preserve the quality of the transplanted organs and tissues.

Therefore, the assessment of tissue harvesting and use should receive more attention. In particular, cornea [31], gall bladder and choledocus [32], osteotendinous tissue, and vessels were harvested from ECMO donors [13], but nothing was shared about their status and fate.

Some promising indications can be identified in the study performed by Constantinescu et al. [34], addressing musculoskeletal tissue. In particular, they underlined how tissue preservation in limb extremities represents an important challenge. The authors were also able to perfuse porcine limbs ex vivo and assess tissue biopsies, demonstrating the potential of extracorporeal blood circulation in preserving muscles, blood vessels, and nerves. However, in that study, the authors implemented a condition of perfusion which was different from the regional ECMO. As a major flaw, the paper did not report information about ligaments, cartilage, or menisci. Indeed, a porcine model was used, but concerning—for instance—meniscal tissue, no known animal species give appropriate substitutes with respect to human tissue [49].

On the other hand, the capacity of bone, tendons, fat, and skin to tolerate ischemia has been already discussed [50,51].

Besides these few references on ECMO tissues in general, there is no evidence of preclinical studies comparing regional ECMO with respect to standard donors. They should be planned and carried out to assess the potentiality of the ECMO donor pool with statistical significance. If positive, clinical studies could then verify the real efficacy of ECMO tissue grafts.

Therefore, before starting to distribute musculoskeletal tissues harvested from ECMO donors, a study procedure should be proposed, starting directly from human donors, standing the non-use of vital organs. Experiments should focus on tissue structure/composition and function, thus, to exclude correlation between potential macroscopic alterations and the extracorporeal oxygenation procedure. The goal of this study should be the possibility to increase the overall number of tissue donors for allograft, thus, to make bone transplantation and joint reconstruction procedures feasible for more patients, guaranteeing, at the same time, the safety and quality of the harvested samples.

Regarding the structure and composition of tendons, histological and immunohistochemical analysis of biopsies are fundamental to provide information concerning cellularity, vascularity, fibrous matrix, and content of glycosaminoglycans (GAGs) [52]. Normal tendons are featured by organized, parallel fibre bundles and long, thin tenocytes dispersed throughout the matrix. On the other hand, pathological tendons may show various alterations, such as glassy appearance, loss of matrix organization, GAG accumulation, or intimal hyperplasia of blood vessels [53].

Although tendons and ligaments have many similarities in terms of structure, the latter shows a higher proteoglycan and water content and a lower amount of collagen. As for tendons, histology and immunohistochemical staining could provide information about collagen bundle width, fibril orientation, cell morphology and size, as well as crimp (i.e., a regular sinusoidal pattern in the matrix of collagen fibres [54]). Regarding menisci, lesions following trauma or related to osteoarthritis (OA) were fully described [55,56], though meniscus properties after ECMO seem to have never been systematically analysed. Therefore, dealing with structure and composition, the study procedure will establish protocols for representative macroscopic and histopathological analysis to exclude the presence of potential proteolytic degradation or other biochemical alterations [57].

Meanwhile, mechanical tests would give indications about function [58,59]. In the case of tendon grafts, the study protocol could be based, for instance, on the work of Zheng et al. [59]: grafts are tested in traction, standing that these tissues are mainly composed of fibres for resisting tension; testing groups are compared in terms of stiffness and ultimate failure at maximum load; they differed for the pretension force, while, in the study on ECMO, they would differ for the kind of donor. Similar discussion applies to meniscus grafts: testing protocol could be similar to that contained in the work of Sandmann et al. [58], where samples are loaded in compression, as in the main physiological functioning, and evaluated in terms of stiffness and residual force; the comparison was between different species and artificial constructs, while, in the study on ECMO, it would be between different kinds of human donor.

Indeed, mechanical testing could alter the sample; therefore, it should not precede histology. On the other hand, histology represents a standard assessment, but preparation is destructive (usually embedding and sections cutting), therefore, it cannot work on the same sample of the mechanical test. It follows that, if biopsy on which histology is performed decreases the mechanical strength of the tissue—mainly due to harvesting dimensions and/or location—two different groups of tissues are needed, one for each assessment (mechanical and histological), with an increase in experimental time and costs.

Finally, by comparing a group of samples harvested from standard donors with respect to a group coming from ECMO donors, it is possible to demonstrate that, statistically—that is on average—the two populations do not differ from each other. Nonetheless, although safety is guaranteed by microbiological testing, the evaluation of each specific graft before implantation remains fundamental [60], especially regarding suitability issues. For what is said above, in the case of musculoskeletal tissues, a biopsy could not be feasible because the process can alter the graft function. Therefore, gross evaluation and instruments sensing mechanical strength without altering the tissue are mandatory. Those instruments could be represented by a ligament tensioner in the case of anterior cruciate ligament grafts, as used in the study of Niki et al. (2019) for second-look arthroscopy [61], or by a blunt tip for sensing fibro-cartilaginous tissue consistency, as proposed by the International Cartilage Repair Society (IRCS) grading system [62].

Once the tissue grafts harvested from ECMO donors are declared to be potentially safe and effective, post-transplant clinical studies will state their effectiveness. As for organs, in vivo assessments should be focused both on implanted patients and on used allografts. In the case of musculoskeletal tissues, the patients could be evaluated through pain and activity scores [63], while grafts by biomedical imaging could be able to provide information about morphology and biochemistry [61,64,65,66].

## 5. Conclusions

*Post-mortem* extracorporeal membrane oxygenation (ECMO) is a potential way to expand the overall number of tissue donors for allografts. In order to verify its feasibility, tissues harvested from ECMO donors must be compared with those coming from standard donors by defining specific ex vivo experiments, which could be represented by histological analyses, and mechanical testing in the case of musculoskeletal tissue grafts.

## Data Availability

No new data were created or analysed in this study. Data sharing is not applicable to this article.

## Supplementary Materials

The following are available online at https://www.mdpi.com/article/10.3390/membranes11070545/s1, Table S1: Summary of specific details of studies included in the synthesis.
